# A Low-Cost, Passive Release Device for the Surveillance and Control of Mosquitoes

**DOI:** 10.3390/ijerph16091488

**Published:** 2019-04-27

**Authors:** Michael W. C. Kwan, Alexander Bosak, Jedidiah Kline, Mario A. Pita, Nicholas Giel, Roberto M. Pereira, Philip G. Koehler, Daniel L. Kline, Christopher D. Batich, Bradley Jay Willenberg

**Affiliations:** 1 Department of Internal Medicine, College of Medicine, University of Central Florida, Orlando, FL 32827, USA; michael.kwan@ucf.edu (M.W.C.K.); alexander.bosak@ucf.edu (A.B.); ngiel06@gmail.com (N.G.); 2 Department of Materials Science and Engineering, University of Florida, Gainesville, FL 32611, USA; Jedidiah.Kline@ucf.edu (J.K.); cbati@mse.ufl.edu (C.D.B.); 3 United States Department of Agriculture-Center for Medical, Agricultural and Veterinary Entomology, Gainesville, FL 32608, USA; mariapita99@gmail.com (M.A.P.); Dan.Kline@ARS.USDA.GOV (D.L.K.); 4 Entomology and Nematology Department, University of Florida, Gainesville, FL 32611-0620, USA; rpereira@ufl.edu (R.M.P.); pgk@ufl.edu (P.G.K.); 5 J. Crayton Pruitt Family Biomedical Engineering, University of Florida, Gainesville, FL 32611, USA

**Keywords:** Spatial repellent, controlled release, surveillance, military, malaria, field-deployable device, Zika

## Abstract

Mosquitoes continue to be a major threat to global health, and the ability to reliably monitor, catch, and kill mosquitoes via passive traps is of great importance. Global, low-cost, and easy-to-use outdoor devices are needed to augment existing efforts in mosquito control that combat the spread of disease, such as Zika. Thus, we have developed a modular, portable, non-powered (passive), self-contained, and field-deployable device suitable for releasing volatiles with a wide range of applications such as attracting, repelling, and killing mosquitoes. This unique device relies on a novel nested wick and two-reservoir design that achieves a constant release of volatiles over several hundred hours. Devices loaded with one of either two compounds, geraniol or 1-methylpiperazine (MP), were tested in a controlled environment (32 °C and 70% relative humidity), and both compounds achieved a constant release from our devices at a rate of 2.4 mg/h and 47 mg/h, respectively. The liquid payload can be volatile attractants or repellants as well as mosquitocide-containing feeding solutions for capture and surveillance. This low-cost device can be utilized for both civilian and military mosquito control purposes, but it will be particularly important for protecting those in economically repressed environments, such as sub-Saharan Africa and Central and South America.

## 1. Introduction

Mosquito-borne infections account for the collective loss of over 50 million years of healthy human life [1]. Associated pathogens include malaria, dengue, chikungunya, and Zika virus. The burden from mosquito-borne disease is expected to increase as global commerce, human travel, and deforestation/urbanization of the tropics continually increase vector distribution and transmission of vector-borne diseases [2]. Malaria alone accounted for 435,000 deaths in 2016, 91% of these occurring in Africa [3]. Of note is the disproportionate effect of vector-borne diseases on poverty-stricken populations [1] with a high correlation between poverty and malaria prevalence [4,5].

From 2001 to 2015, insecticide-treated nets (ITNs) and indoor residual spraying (IRS), the main non-pharmaceutical mosquito interventions, have prevented an estimated 457 million and 66 million cases of malaria, respectively [4]. Despite these successes, ITNs and IRS do not reach all vulnerable populations. In sub-Saharan Africa, an estimated 60% of households at risk for malaria either do not have access to ITNs, or do not have enough ITNs for all occupants [3]. Furthermore, IRS coverage is declining, with utilization by only 6.6% (64 million) of those at risk in this region [3]. Insecticide resistance [5,6,7,8] and shifts in mosquito feeding/resting behaviors (i.e., indoor to outdoor), possibly in response to ITNs and IRS, further exacerbate these deficiencies and allow transmission to persist even in high ITN and IRS coverage areas [9,10,11]. Therefore, novel strategies that are low-cost and easily accessible are needed to bolster existing control strategies.

Controlled-release devices are widely used, critical components for mosquito control studies and strategies. These devices release volatiles into the air to either repel mosquitoes from an area or attract them into traps [12]. Emanator-type controlled release devices release volatile payloads through evaporation (either powered or passive) and are categorized by either zero-order or first-order release kinetics. For zero-order, the release rate is independent of the payload amount or concentration and is constant over time. In contrast, first-order release is dependent upon instantaneous payload concentration and has a decreasing release rate over time. Further development and analysis of the release kinematics of spatial repellants from passive devices is crucial to the development of cost-effective devices. Here we describe a low-cost, easy-to-use device capable of utilizing liquid payloads for various mosquito management strategies. Previously, 1-methylpiperazine [13] and geraniol [14] have been shown to act as volatile repellants. We have chosen to focus on these two repellants to test our passive release device in a humidified environment.

**Figure 1:**
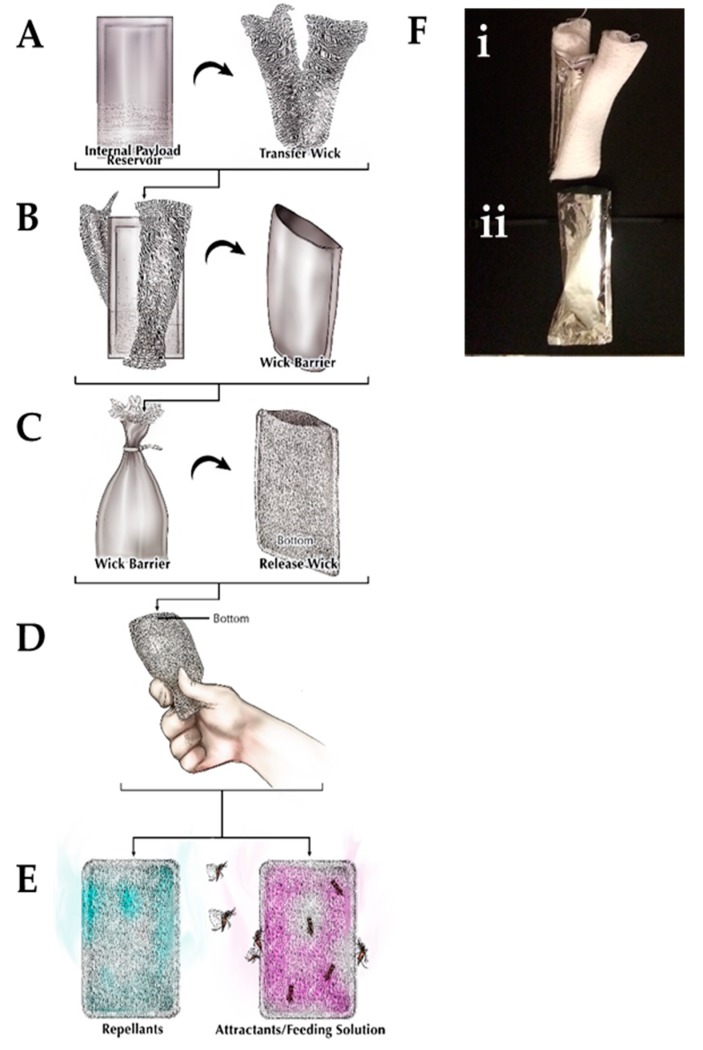
Controlled-release device. The (**A**) internal payload reservoir is surrounded by the transfer wick and they are placed together inside the (**B**) wick barrier. This wick barrier is placed inside the (**C**) release wick before being (**D**) utilized to (**E**) release either repellants or attractants/feeding solutions. (**F**) Actual images of the (i) internal reservoir and transfer wick that is inserted into the (ii) wick barrier.

In contrast to currently marketed devices, which rely on active emanation of repellants via a fan or heat source, such as the Terminix AllClear Sidekick^TM^, SC Johnson’s OFF!^®^ Clip-On^TM^, or Therma-CELL^®^ [15], our device is completely passive and is only composed of cotton and aluminized polymer film (polyethylene and nylon) (Figure 1). The inner reservoir can store a variety of active ingredients (AIs) and is impermeable to commonly used spatial repellents and insecticides. This allows the device to be stored and handled without releasing the AI prior to use. Once ruptured by the end user with a simple hand squeeze, the AI moves to the outer reservoir, which contains a wick that controls the release rate (Figure 1D). These characteristics, combined with our dual reservoir technology, make our device, to the best of our knowledge, unique [16].

## 2. Materials and Methods 

This device was comprised of an aluminum nylon and polyethylene balloon, cotton gauze, a cable tie, and cotton terrycloth. The internal payload reservoir (Figure 1A) was constructed from an 8.5 cm × 11 cm piece of balloon material, which was folded along the long side and heat-sealed creating an open pouch on the short side. To improve payload deployment and reservoir rupture, a weak point was created in the payload reservoir by scoring the desired location with 400 grit sandpaper (Figure 1A). The solution of interest (15 mL) was added to the internal payload reservoir, and the open end was closed and heat-sealed (excess film was removed after sealing). A 10 cm × 20 cm cotton gauze transfer wick was folded around the internal payload reservoir (Figure 1B). The wick barrier was constructed from a 10 cm × 10 cm piece of balloon material, which was folded in half and heat-sealed creating an open pouch. The internal payload reservoir with the transfer wick was inserted into the wick barrier (Figure 1B). A 10 cm cable tie was then tightened around the open end of the wick barrier, allowing the transfer wick to extend beyond the wick barrier (Figure 1C). The releasing wick was constructed from a 24 cm × 12 cm piece of cotton terrycloth, which was folded in half along the long side and sewn into an open pouch. The wick barrier was inserted into the releasing wick, and the open end was sewn shut (Figure 1C). To activate the device, the end-user squeezed the whole device from the cable tie end, thereby rupturing the internal payload reservoir through the scored section and releasing the stored solution (Figure 1D,E). The average time needed to construct a device was about ten (10) min.

To test the release of volatiles from our device, two compounds that altered insect behavior, geraniol (Sigma-Aldrich) and 1-methylpiperazine (1-MP, Sigma-Aldrich Lot # STBF1692V) were utilized. A 15 mL (13.3 g) amount of each compound was placed in separate devices and allowed to evaporate inside a controlled, humidified environment (32 °C, 70% relative humidity) for 600 h. The humidified environment was under 2.5 L/min airflow and complete air exchange was approximately every 14 h. Devices were weighed at the beginning of the experiment and repeatedly measured (technical replicates) throughout the experiment to assess the amount of volatile agent that had been released. Experiments were carried out a minimum of three (3) times for each unique AI (biological replicates). Standard deviations were calculated and linear regression analyses were used to model the constant release of volatiles as a function of time (i.e., mass vs. time); these analyses were performed in Microsoft Excel.

## 3. Results

**Figure 2:**
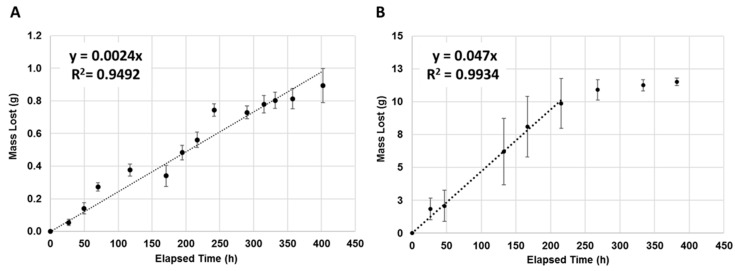
Release of geraniol and 1-methylpiperazine (1-MP) from passive wick and reservoir devices. Regression analyses of the release rate of (**A**) geraniol and (**B**) 1-MP showed zero order release (R^2^ = 0.9492 and 0.9934, respectively). Regression analyses were calculated over the first 400 and 215 h, respectively. Samples were measured within a controlled environment: 32 °C and 70% relative humidity.

Both volatiles exhibited zero-order release from our device with R^2^ values of 0.9492 and 0.9934 for geraniol and 1-MP, respectively (Figure 2). 1-MP was released at a faster rate of 47 mg/h resulting in a nearly depleted device within 215 h, as shown by the plateau regime in Figure 2B, and the regression line was only fitted to the first 215 h. Meanwhile, geraniol released at a rate of only 2.4 mg/h and after 400 h still had ~94% of the total reservoir mass remaining. Subsequently, the regression line was fit to the entire elapsed time suggesting that the geraniol devices may have still been in the process of releasing its payload. This difference in behavior between the two compounds was expected, as 1-MP is more volatile than geraniol with a boiling point at 138 °C compared to geraniol’s boiling point of 230 °C. Because these compounds exhibited zero-order release kinetics at some point in the experiment, it was expected that altering the exposed wick area would alter the linear-release lifetime of the device, as shown for other controlled-release systems [17,18].

Data were collected after the 450 h time point for both formulations; however, the geraniol release data became erratic and unreliable because of suspected instabilities in environmental chamber conditions. Meanwhile, the 1-MP devices essentially stopped emanating after 450 h because ~90% of the 1-MP payload was released. We believe that the remaining unspent payload became trapped in folds and pockets created as the film walls of the payload reservoir collapsed during the lifecycle of the device. This trapped 1-MP likely had insufficient contact with the transfer wick to be carried to the release wick, and it ultimately volatilized. This contention was supported by the observation of remnant liquid in the reservoirs of spent/used devices.

## 4. Discussion

The controlled release devices in this investigation had a constant release of two known mosquito repellents over a 215 h study period. Assuming that the wick of our device was fully saturated and there was liquid in the reservoir, the release rate should continue to be constant. Over the time period that a constant rate of release is attained, the device is said to be in the zero-order release regime. A zero-order release is desirable for maintaining an effective airborne concentration of the spatial repellent independent of time and remaining reservoir mass. The release rate will be a function of many variables including the volatility of the payload formulation, the evaporation rate of the formulation from the wick material surface, and the effective exposed surface area of the wick. In practice, for a given payload formulation, set of experimental conditions (i.e., temperature and humidity), and wick material, the release rate of wick-in-reservoir devices can be changed by altering the exposure surface area of the wick. The zero-order release behavior described above only applies to pure, single component payload formulations; the release rates for multicomponent payload formulations will change with time, because of differences in vapor composition when compared to the liquid-payload composition, unless azeotropic formulations are used. Although attractants were not tested in this study, it was hypothesized that the release of homogenous attractants from the device would follow the constant release behavior, which may be useful for a “push” and “pull” mosquito control technique [13,19].

The study of geraniol-loaded devices demonstrated that slow release rates (2.4 mg/h) were achievable. Such a slow release corresponds with low airborne concentrations and may be useful for active ingredients that are effective in low airborne concentrations. When compared to other passive release devices comprised of a fabric impregnated with a volatile [20,21], the release rate of the device presented here will likely be lower due to restricted release of the volatile in the inner reservoir through the transfer wick. The period of time that the device releases active ingredients in a constant, zero-order manner is also anticipated to be relatively longer due to this intentional slowing. This slower release may be useful to extend the lifetime of the device and would help reduce the frequency of device replacement, a feature that is particularly useful in developing, low-income regions and desolate areas.

The release rate of the device may vary in a semi-field or full-field test as the different boundary conditions (i.e., the total volume studied) may alter the rate of volatiles leaving the device. Compared to our enclosed environmental chamber, the in-field airborne concentration will be less as the boundary condition can be approximated as an infinite well (i.e., the atmosphere), effectively diluting the released mass [22]. Additionally, we postulated that the release rate would increase as the concentration gradient between source (device) and sink (environment) increased in the semi-field or full-field tests, as governed by Fick’s law of diffusion. The resultant increase in release rate would lessen the time the device would be emanating volatiles, as the reservoir would be depleted faster. This effect can be ameliorated by using an adjustable external membrane or container to reduce the exposed surface area of the device.

Field testing of this device is needed to confirm that the observed laboratory results are representative of field performance and to confirm the behavioral effects on mosquitoes. To test field performance, the device could be weighed at activation and again after some predetermined elapsed time. Airborne concentration measurements of the active ingredient via air sampling followed by thermal desorption-gas chromatography mass spectrometry [23] may be used as another method to compare the laboratory and field performances of the device. Finally, trapping studies that quantify mosquito catch rates in semi-field or full-field conditions could serve as a testing platform to assess the effects of the device on mosquito behavior. Under semi-field conditions, a known number and age of several different mosquito species could be released, and the catch rates of traps with and without active ingredient-loaded devices could be compared. Variables to be studied include the active ingredient used, time of device deployment, distance between trap and device, age, sex, and species of mosquitoes.

## 5. Conclusions

In this work we describe a passive release device capable of achieving a constant rate of release of spatial repellents over approximately nine (9) days. This passive wick device will be helpful in mosquito control as it allows for predictable release of repellant compounds without the need for electrical power or high-tech devices. An end-user would only need to break the pouch and hang the volatile- or attractant-containing pouch either by itself or within a trap. This variable release, low-cost, passive device is readily implementable in the field by mosquito control professionals, and it may find use in civilian and military sectors alike.

## 6. Patents

Willenberg, B.J., Koehler, P.G., Batich, C., Georgiades, G., inventors. Methods and devices for sustained release of substances. USA 9,258,988. 2015 January 22.
